# A New Genus and Species of Cochliopidae Tryon, 1866 (Truncatelloidea) from the Dominican Republic

**DOI:** 10.3390/ani16121810

**Published:** 2026-06-11

**Authors:** Andrzej Falniowski, Aleksandra Jaszczyńska, Sebastian Hofman

**Affiliations:** 1 Department of Malacology, Institute of Zoology and Biomedical Research, Jagiellonian University, 30-387 Kraków, Poland; 2 Department of Invertebrate Evolution, Institute of Zoology and Biomedical Research, Jagiellonian University, 30-387 Kraków, Poland; a.jaszczynska@uj.edu.pl; 3 Department of Comparative Anatomy, Institute of Zoology and Biomedical Research, Jagiellonian University, 30-387 Kraków, Poland; s.hofman@uj.edu.pl

**Keywords:** morphology, cytochrome oxidase, *16S*, *12S*, *18S*, molecular phylogeny, integrative taxonomy

## Abstract

A new genus and species of the Cochliopidae are described from the Dudu cenote, Dominican Republic, Hispaniola Island. Shell and its measurements, soft parts morphology and pigmentation, radula, female reproductive organs, and penes are described. Mitochondrial cytochrome oxidase subunit I (*COI*), *16S* and *12S*, and nuclear *18S* sequences have been applied to reconstruct phylogenetic relationships. The Hispaniolan taxon clearly belongs to a clade that also includes many *Heleobia* W. Stimpson, 1865, species from the Americas, the European *Eupaludestrina* Mabille, 1877, as well as North American *Heleobops* F. G. Thompson, 1968. All analyses support the assignment of the Hispaniolan taxon to a distinct genus, new to science. Its known geographic distribution is restricted to this cenote so far.

## Simple Summary

Antilles Islands inhabit interesting freshwater snails’ fauna, shaped by the geological history of the archipelago, isolation, and migrations. This fauna, however, is still poorly studied. At Hispaniola Island, in the Dominican Republic, in the Dudu cenote, a small pond originated by partial collapse of a cave, some minute snails were found in 2001, representing the Truncatelloidea, the most diverse and species-rich, globally distributed superfamily of fresh- and brackish-water gastropods. Both morphological and molecular (DNA) characters showed that these animals represent the family Cochliopidae but are as distinct as to be classified as new to science, both as a species and a genus. The genus is named in honour of Dr Robert Hershler, a highly merited malacologist devoted to the study of the Cochliopidae. These snails still inhabit this pond.

## 1. Introduction

In the Dominican Republic (Hispaniola Island), Greater Antilles, 14 species of freshwater gastropods have been recorded so far [1,2,3,4,5,6,7]: nine species represent Caenogastropoda, and five—Hygrophila. In the Antilles, the superfamily Truncatelloidea J. E. Gray, 1840, is represented by the family Cochliopidae Tryon, 1866. Two species of the genus *Nanivitrea* Thiele, 1927, have been recorded from Cuba, *N. alcaldei* Jaume and Abbot, 1947, and *N. helicoides* (Poey, 1865) [8], and another two from Jamaica, *N. inconspicua* (C. B. Adams, 1851) and *N. pygmaea* (C. B. Adams, 1849). These low-spired, valvatoid gastropods, initially assigned to the Assimineidae H. Adams et A. Adams, 1856 [9], were later transferred to the Cochliopidae [10,11,12]. *Pyrgophorus* Ancey, 1888, is another cochliopid genus reported from this area—from Venezuela [13,14,15] (six species), Guadalupe [16,17] (two species), and Martinique [18] (one species). In Venezuela and the latter two islands, *Pyrgophorus parvulus* (Guilding, 1828) has been found. Pfeiffer [19] described *Paludina coronata* L. Pfeiffer, 1840, from Cuba, later assigned to the genus *Pyrgophorus* and recorded from several localities in Cuba [20].

**Figure 1:**
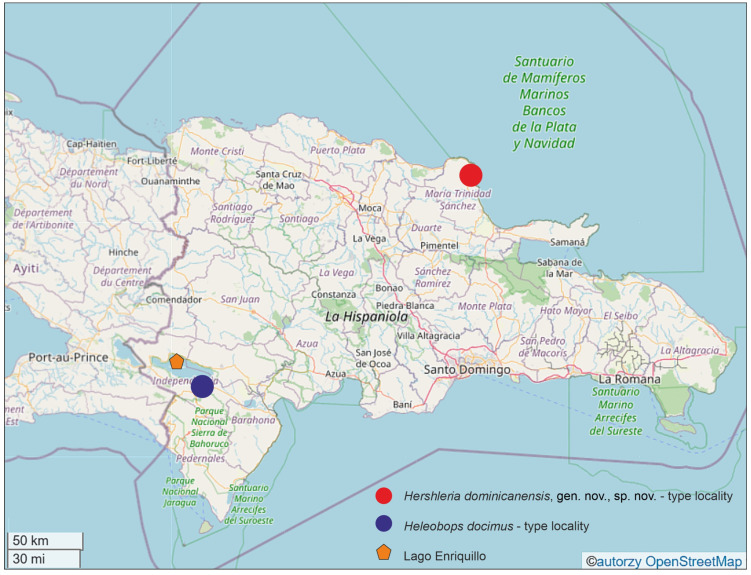
Map showing localities of *Hershleria dominicanensis* and other representatives of Cochliopidae in the Dominican Republic. The map is based on OpenStreetMap data, and all locality names are presented in their original forms.

On Hispaniola Island, *P. coronatus* has been recorded from Lake Miraguane in the western part of Haiti [2], and on the same island, in the Dominican Republic, *P. coronatus bermudezi* (Aguayo, 1947) has been found in the Pleistocene sediments, near Lake Enriquillo (Figure 1) [21]. In the Dominican Republic, another representative of the Cochliopidae, genus *Heleobops* F. G. Thompson, 1968, has been found [6]: *Heleobops clytus* F. G. Thompson et Hershler, 1991 (now assigned, as a junior synonym, to *H. docimus* F. G. Thompson, 1968 [22]), reported from 2 km ESE of Duvergé (Figure 1), Independencia Province, Lake Enriquillo basin, and spring Boca de Cachón. *Antilobia margalefi* Altaba, 1993, was described from Lake Enriquillo [23], the taxon later synonymized with *Pyrgophorus coronatus* bermudezi [22]. *Heleobops docimus* inhabits also a pond in the Cayman Islands [24]. *Heleobops torquatus* F. G. Thompson et Hershler, 1991, has been found on Jamaica [6].

In the materials deposited in the Zoological Museum of the Jagiellonian University by an anonymous collector there was a jar containing a few minute truncatelloid snails collected in the Dudu Blue Lagoon cenote, Dominican Republic, in April 2001. Recently, on February 27th, 2025, we inspected this cenote (Figure 1) and confirmed the presence of these snails there. The Dudu cenote is recently a protected area; thus, we did not collect these snails then, and the entire study is based entirely on these old museum materials. The snails represent the family Cochliopidae. In the paper, we present the morphology and the molecularly inferred phylogenetic relationships of this new taxon.

## 2. Materials and Methods

**Figure 2:**
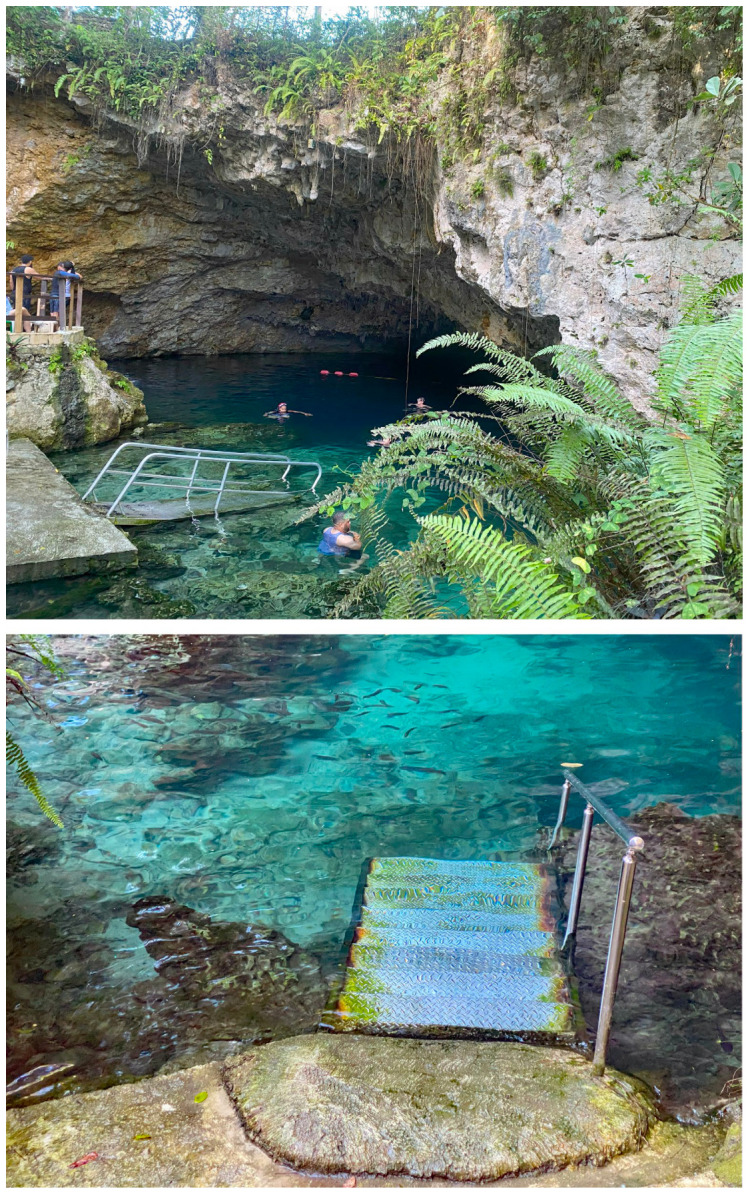
Type locality of *Hershleria dominicanensis*, Dudu cenote, María Trinidad Sánchez Province, Dominican Republic.

The snails still occur on a delicate layer of filamentous algae growing on stones, at a depth of approximately 0.5 m, in the Dudu Blue Lagoon cenote, 19°38′51.0″ N 70°04′38.9″ W, near Cabrera, María Trinidad Sánchez Province, Hispaniola Island, Dominican Republic, at the eastern end of the country’s northern coast (Figure 1). A cenote is a natural pit or sinkhole, resulting from the collapse of limestone bedrock that exposes groundwater underneath. The main lake, 16 m deep, is partially covered by a ceiling with stalactites. Underground branches of the cave run radially from the lake and are 90–135–350 m long. The temperature of the water ranges between 25 and 27 °C. Like in most limestone-based cenotes, the water is neutral to slightly alkaline and slightly brackish. Without permission, we were not able to analyse the water chemistry in this protected area. The snails inhabit the light zone (Figure 2) and show no trace of troglomorphism. The snails were kept in 80% analytical ethanol and stored at −20 °C.

The shells were photographed with a Canon EOS 50D digital camera, under a Nikon SMZ18 microscope. The dissections were done under a Nikon SMZ18 microscope with dark-field, equipped with a Nikon DS-5 digital camera, whose captured images were used to draw female reproductive organs with a graphic tablet. Morphometric parameters of the shell were measured by one person using ImageJ v. 1.53g image analysis software [25]. Protoconchs were cleaned using an ultrasonic cleaner, mounted and examined, and the radulae were extracted with Clorox, all applying the techniques described by Falniowski [26]. Protoconchs and radulae were photographed using a Hitachi S-4700 scanning electron microscope. Penes were photographed under a Motic B3 Professional microscope with dark field. Morphological terminology after Hershler and Ponder [27].

DNA was extracted from whole specimens; tissues were hydrated in Tris-EDTA (TE) buffer (3 × 10 min); then, total genomic DNA was extracted with the Sherlock extraction kit (A&A Biotechnology, Gdańsk, Poland), and the final product was dissolved in 20 μL of TE buffer. The extracted DNA was stored at −80 °C at the Department of Malacology, Institute of Zoology and Biomedical Research, Jagiellonian University in Kraków (Poland).

Mitochondrial cytochrome oxidase subunit I (*COI*), *16S* ribosomal RNA (*16S*), and *12S* ribosomal RNA (*12S*) were sequenced. Details of PCR conditions, primers used, and sequencing were given in Szarowska et al. [28] and Collado et al. [29]. Phylogenetic analyses using *COI* were performed by adding sequences from GenBank (Table 1), available for the representatives of all the cochliopid genera inhabiting Central America, the Antilles, the northern part of South America, and the southern part of the USA. The representatives of the genera *Aphaostracon* J. G. Thompson, 1968, *Cochliopa* W. Stimpson, 1865, *Cochliopina* J. P. E. Morrison, 1946, *Littoridina* Souleyet, 1852 [30], *Littoridinops* Pilsbry, 1952, *Tryonia* Stimpson, 1865 [31], as well as widely distributed but geographically far *Heleobia* W. Stimpson, 1865, were included in the *COI*-based analyses. Unfortunately, from among the geographically close genera, there were no sequences of *Emmericiella* Pilsbry, 1909, *Subcochliopa* J. P. E. Morrison, 1946, and *Texadina* Abbott & Ladd, 1951. There were no sequences of the representatives of these genera in GenBank, and we were unable to obtain these specimens for sequencing.

Sequences were initially aligned using MUSCLE [32] software, implemented within MEGA 7 [33], and then visually checked in BioEdit 7.1.3.0 [34]. Uncorrected *p*-distances were calculated in MEGA 7. The estimation of the proportion of invariant sites and the saturation test for entire data sets [35,36] were performed using DAMBE [37]. The data were analysed using Bayesian inference (BI) and maximum likelihood (ML), in MrBayes v. 3.2.7a [38] and RAxML-NG v. 0.8.0 [39], respectively. The appropriate substitution model for ML analysis was selected using jModelTest2 via the CIPRES Science Gateway [40]; the model TPM2uf+I+G was used. Partitioning of the data into three codon positions for *COI* was performed. The ML analysis was conducted in RAxML-NG v. 0.8.0 via the web service available at https://raxml-ng.vital-it.ch/ (accessed on 20 March 2025), with 10 random and 10 parsimony-starting trees. In the BI analysis, the K81+G model of nucleotide substitution was applied in tree reconstruction. The substitution model was selected using MrModelTest 2.4 [41]. The analyses were run using MrBayes v. 3.2.7a [38] with default priors. Two simultaneous analyses were performed, each with 10,000,000 generations, with one cold chain and three heated chains, starting from random trees and sampling the trees every 1000 generations. The first 25% of the trees were discarded as a burn-in. The analyses were summarized as a 50% majority-rule consensus tree. Convergence of the runs was checked in Tracer v.1.7.1 [42], and all effective sample sizes exceeded 200, the proposed measure of good sample mixing. Fig Tree v. 1.4.4 [43] was used to visualize the trees. Two species delimitation methods were performed: Poisson Tree Processes (PTPs) [44] and Assemble Species by Automatic Partitioning (ASAP) [45]. The PTP approach was run on the web server (https://species.h-its.org/ptp/ accessed on 20 March 2025), with 100,000 MCMC generations, 100 thinning intervals, and 0.1 burn-in. We used the RAxML output phylogenetic tree. The ASAP approach was run on the web server (https://bioinfo.mnhn.fr/abi/public/asap/, accessed on 20 March 2025) using simple distance (*p*-distances). The Fastachar application [46] and DeSignate [47] were used to determine the Molecular Diagnostic Characters (MDCs) for *COI*. Two types of characters were accepted: at binary positions (where the character state in the query group is different from the uniform character state of the reference group) and at asymmetric positions (where the character state in the query group is not different from the uniform character state of the reference group). The letters used in the MDCs list denote the nucleotide position in our sequence set, and the letter in parentheses denotes the specific nucleotide characteristic for the particular group. These analyses were performed on the set of sequences shown in the Supplementary Materials (Supplementary File S1).

**Table 1:** Reference sequences used in phylogenetic analyses.

Species	*COI* GB Numbers	References
*Aphaostracon* sp.	AF129319 AF129320	Hershler et al. 1999 [24]
*Aroapyrgus* sp.	AF354759	Liu et al. 2001 [48]
*Cochliopa* sp.	AF354762	Liu et al. 2001 [48]
*Cochliopina riograndensis* (Pilsbry and Ferriss, 1906)	OR372132	Perez et al. 2023 [49]
*Eupaludestrina aponensis* (E. von Martens, 1858)	JQ973021	Kroll et al. 2012 [50]
*Eupaludestrina dobrogica* (Grossu and Negrea, 1989)	EU938132	Falniowski et al. 2008 [51]
*Eupaludestrina maltzani* (Westerlund, 1886)	KM213723	Szarowska et al. 2014 [52]
*Eupaludestrina scamandri* (Boeters, Monod, and Vala, 1977)	JQ973025	Kroll et al. 2012 [53]
*Eupaludestrina stagnorum* (Gmelin, 1791)	JQ973024	Kroll et al. 2012 [53]
*Heleobia ascotanensis* (Courty, 1907)	MF447993 OQ847325	Valladares et al. 2018 [54] Valladares et al. 2024 [55]
*Heleobia atacamensis* (R. A. Philippi, 1860)	OP630467	Valladares et al. 2024 [55]
*Heleobia australis* (A. d’Orbigny, 1835)	JQ972708 MT295136	Kroll et al. 2012 [53] Unpublished
*Heleobia carcotensis* Collado, Valladares, and Méndez, 2016	KR816827	Collado et al. 2016a [56]
*Heleobia charruana* (A. d’Orbigny, 1841)	MW717674	van Haaren et al. 2021 [57]
*Heleobia chimbaensis* (Biese, 1944)	KF658141 MN921148	Collado et al. 2013 [29] Collado et al. 2020 [58]
*Heleobia culminea* (A. d’Orbigny, 1838)	MN094463	Unpublished
*Heleobia* cf. *cumingii* (A. d’Orbigny, 1835)	JQ973041	Kroll et al. 2012 [53]
*Heleobia deserticola* Collado, 2015	KR870998	Unpublished
*Heleobia kuesteri* (Strobel, 1874)	KM220904	Koch et al. 2015 [59]
*Heleobia lacustris* (F. Haas, 1955)	MN094494	Unpublished
*Heleobia languiensis* (F. Haas, 1955)	MN094522	Unpublished
*Heleobia limariensis* (Biese, 1944)	JQ973043	Kroll et al. 2012 [53]
*Heleobia loaensis* (Biese, 1947)	JQ973045 KF658150	Kroll et al. 2012 [53] Unpublished
*Heleobia opachensis* (Biese, 1047)	KF658072	Unpublished
*Heleobia parchappii* (A. d’Orbigny, 1835)	JQ972709 KM220907	Kroll et al. 2012 [53] Koch et al. 2015 [59]
*Heleobia peralensis* Collado, Fuentealba, Cazzaniga, and Valladares, 2020	MN921143	Collado et al. 2020 [58]
*Heleobia piscium* (A. d’Orbigny, 1835)	KM220906	Koch et al. 2015 [59]
*Heleobia poopoensis* (Bavay, 1904)	JQ973050 MN094525	Kroll et al. 2012 [53] Unpublished
*Heleobia transitoria* (Biese, 1947)	KR870995	Unpublished
*Heleobia* sp.	KF658185 KM220908 MH729608	Unpublished Koch et al. 2015 [59] Collado et al. 2019 [60]
*Heleobops carrikeri* G. M. Davis and McKee, 1989	AF213347	Wilke et al. 2000 [61]
*Heleobops docimus* F. G. Thompson, 1968	AF129322	Hershler et al. 1999 [24]
*Littoridinops monroensis* (Frauenfeld, 1863)	EF490565	Hershler et al. 2007 [62]
*Littoridinops tenuipes* (Couper, 1844)	EF490567 OQ918605	Hershler et al. 2007 [62] Unpublished
*Onobops jacksoni* (Bartsch, 1953)	AF367645	Wilke et al. 2001 [63]
*Paladilhiopsis grobbeni* Kuščer, 1928	MH720991	Hofman et al. 2018 [64]
*Pyrgophorus platyrachis* F. G. Thompson, 1968	AF367632 KT372180	Wilke et al. 2001 [63] Ng et al. 2016 [65]
*Strobelitatea hatcheri* (Pilsbry, 1911)	KM220905	Koch et al. 2015 [59]
*Tryonia infernalis* Hershler, H.-P. Liu, and J. S. Simpson, 2015	KP899918	Hershler et al. 2015 [66]
*Tryonia julimesensis* Hershler, H.-P. Liu, and Landye, 2011	JF776789	Hershler et al. 2011 [67]
*Tryonia molinae* Hershler, H.-P. Liu, and Landye, 2011	JF776803	Hershler et al. 2011 [67]
*Tryonia porrecta* (Mighels, 1845)	AY803034	Hershler et al. 2005 [68]
*Tryonia seemani* (Frauenfeld, 1863)	JF776790	Hershler et al. 2011 [67]

## 3. Results

### 3.1. Molecular Phylogeny

**Figure 3:**
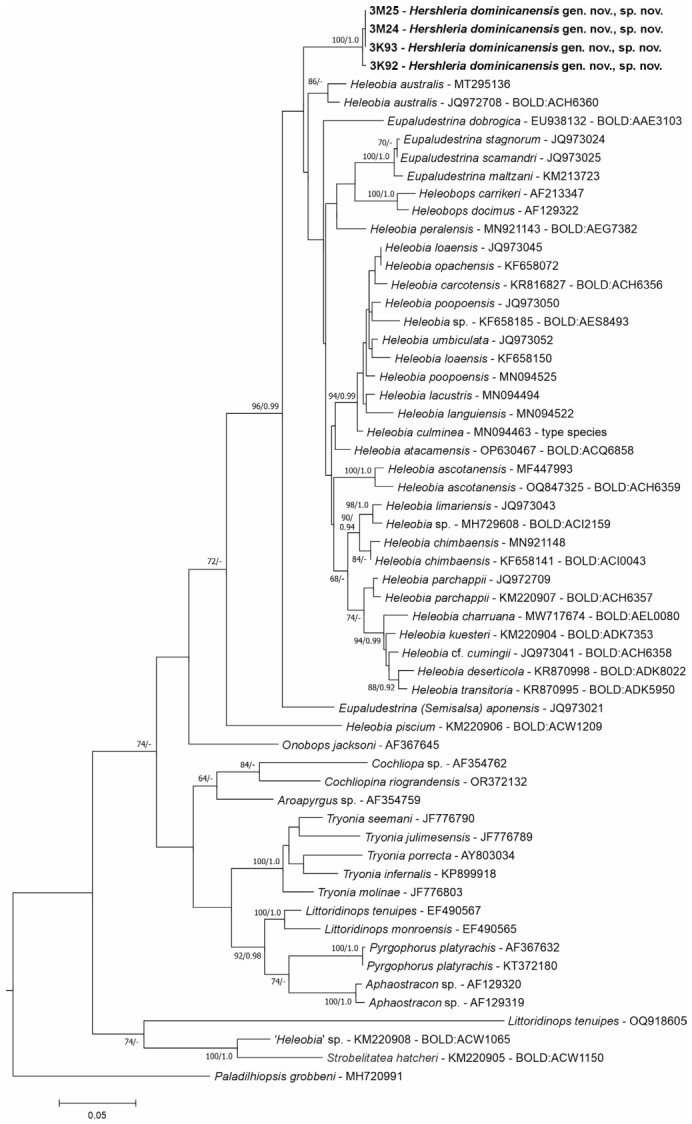
Maximum-likelihood tree computed for *COI*; bootstrap supports over 60%, and Bayesian probabilities over 0.90 are given.

We obtained four new sequences of *COI* (551 bp, GenBank accession numbers PZ349653-PZ349656). The test for substitution saturation analysis showed an ISS value (0.74) significantly below the critical value (ISSC: 0.95), indicating that sequences are not saturated and are therefore suitable for phylogenetic reconstruction. The topologies of the resulting phylograms were identical in both the ML and BI phylogram analyses; thus, we present the phylogram computed with RAxML (Figure 3). All algorithms used for species delimitation supported the distinctiveness of the Dominican snails. The *COI* sequences unambiguously (bootstrap support 100%) placed the Dominican specimens within one Marker gene Operational Taxonomic Unit (mOTU).

This mOTU belonged to the highly supported clade (bootstrap 96%, BP 0.99) of *Heleobia* W. Stimpson, 1865 ([69] type species *Paludina culminea* A. d’Orbigny, 1838), *Eupaludestrina* Mabille, 1877 (type species *Hydrobia macei* Paladilhe, 1867), and *Heleobops* (type species *H. docimus* F. G. Thompson, 1968), and our Dominican taxon. Within this clade, one subclade grouped most of *Heleobia*, another one included *Heleobops* and European/circum-Mediterranean *Eupaludestrina*. In addition, the three genera constituted quite distinct clades (Figure 3). All the genera whose ranges were geographically close to Hispaniola were grouped outside this clade. The *p*-distances between the Dominican cochliopid snail and the other two geographically most closely related cochliopid taxa were 7.4% for Dominican *Heleobops docimus*, and 15.2% for *Pyrgophorus platyrachis* F. G. Thompson, 1968 from Florida. Considering the genetic distances, the closest to our taxon was *Heleobia australis* (A. d’Orbigny, 1835) from Argentina (*p*-distance = 5.3%), although these two taxa did not form a monophyletic group in the *COI* tree, and a large unresolved polytomy was observed there. As shown in the *COI*-based phylogram (Figure 3), these distances are similarly high as the ones between the other genera of the Cochliopidae.

**Figure 4:**
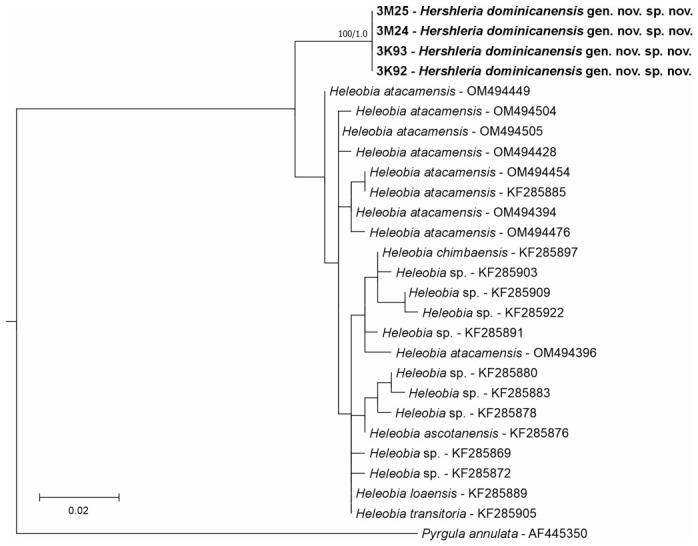
Maximum-likelihood tree computed for *12S*; bootstrap supports over 60%, and Bayesian probabilities over 0.90 are given. The GB numbers of the sequences obtained by Valladares et al. [70] and Collado et al. [29] are also shown.

**Figure 5:**
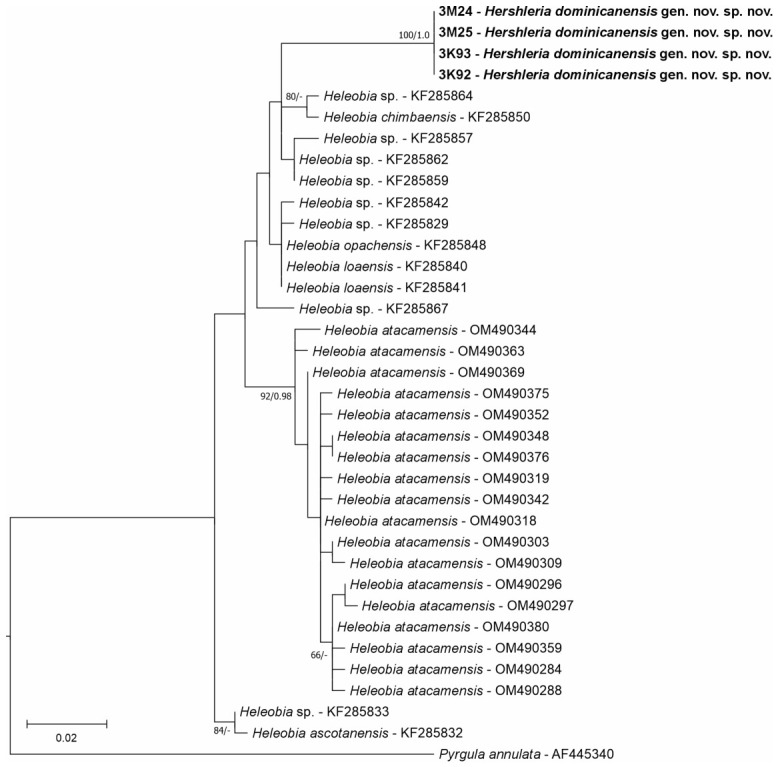
Maximum-likelihood tree computed for *16S*; bootstrap supports over 60%, and Bayesian probabilities over 0.90 are given. The GB numbers of the sequences obtained by Valladares et al. [70] and Collado et al. [29] are also shown.

We also obtained four new sequences of *12S* (311 bp, GenBank accession numbers PZ349056-PZ349059), four new sequences of *16S* (323 bp, GenBank accession numbers PZ349060-PZ349063), and one *18S* sequence (GenBank accession number PZ349601). The number of cochliopid taxa for which mitochondrial *16S* and *12S* sequences are available is very restricted, but our analyses placed the Dominican taxon far from the other *Heleobia* (Figure 4 and Figure 5). All algorithms used for species delimitation also supported the distinctiveness of this taxon.

Considering our phylogeny analyses, species delimitations, as well as geography -thousands of kilometres between Hispaniola and the northern limit of the range of *Heleobia* (Amazonia in South America), the most justified solution seems to describe a new genus for our Dominican taxon.

### 3.2. Systematic Part

Gastropoda Cuvier, 1795

Caenogastropoda Cox, 1960

Littorinimorpha Golikov and Starobogatov, 1975

Truncatelloidea Gray, 1840

Cochliopidae Tryon, 1866

*Hershleria* Falniowski et Jaszczyńska, gen. nov.

ZooBank number: urn:lsid:zoobank.org:act:1000A30E-8FD9-45B0-8E88-0A5786BAFB80

**Type species** *Hershleria dominicanensis* Falniowski et Jaszczyńska, sp. nov., by original designation.

**Diagnosis**

Minute cochliopid snail with conic, moderately broad shell with relatively low spire, whorls slightly convex, ovoid moderately broad aperture, parietal lip complete, radula with one pair of basal cusps and six broad cusps on the cutting edge of central tooth, with big bursa copulatrix closely overlapping on albumen gland, with long duct, long and worm-shaped receptaculum seminis with no distinct duct, sperm tube short, opening into posterior end of pallial cavity, and penis long, flattened, tapering distally, with 5–6 stout, short-stalked apocrine glands similar in size, small and slim non-glandular outgrowth on the right side.

**Etymology**

The genus is named in honour of Dr Robert Hershler, a highly merited malacologist devoted to the study of the Cochliopidae.

**Differential diagnosis**

As for *Hershleria dominicanensis* Falniowski et Jaszczyńska, sp. nov. for this monotypic genus.

***Hershleria dominicanensis*** **Falniowski et Jaszczyńska, sp. nov.**

ZooBank number: urn:lsid:zoobank.org:act:E95B08FE-BFCA-491A-B3F8-4AC5B77F4743

GenBank sequence numbers for *COI*: PZ349653-PZ349656

**Etymology:** *dominicanensis* derived from the Dominican Republic, harbouring the type locality.

**Type locality:** Dudu Blue Lagoon cenote, 19°33′51.9″ N 69°54′28.8″ W, near Cabrera, María Trinidad Sánchez province, Hispaniola Island, Dominican Republic (Figure 1 and Figure 2), on a delicate layer of filamentous algae growing on the stones, at a depth of approximately 0.5 m.

**Figure 6:**
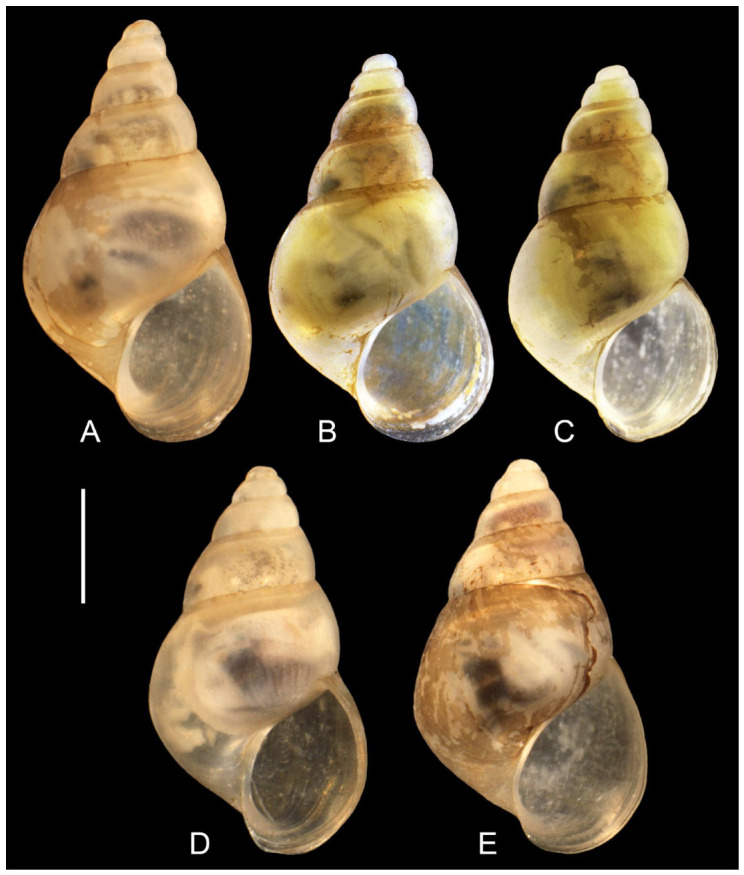
Shells of *Hershleria dominicanensis*: (**A**)—holotype, (**B**–**E**)—paratypes; bar equals 1 mm.

Holotype: Ethanol-fixed specimen (Figure 6A) collected by an anonymous collector, at Dudu Blue Lagoon cenote, 19°33′51.9″ N 69°54′28.8″ W, Hispaniola Island, Dominican Republic, April 2001, deposited in the Zoological Museum of the Jagiellonian University, voucher number ZMUJ/2026/07.

Paratypes: Seven ethanol-fixed specimens collected at the type locality, voucher number ZMUJ/P/08–13, two paratypes destroyed for DNA extraction, three others for dissection.

**Description**

**Figure 7:**
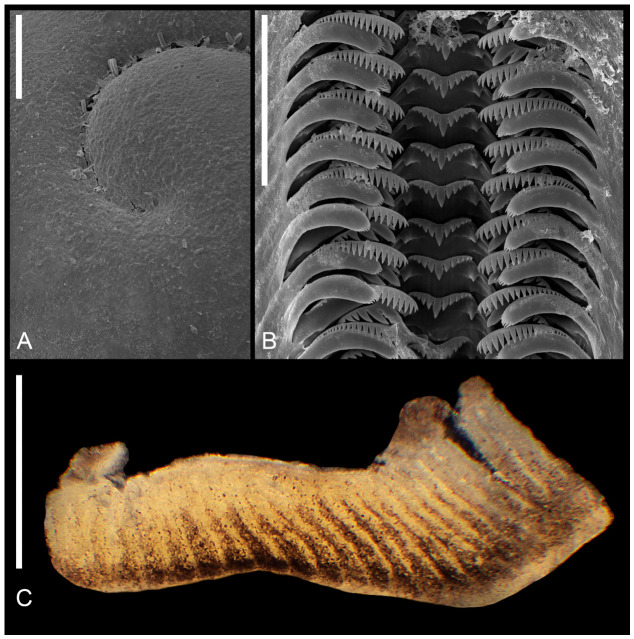
*Hershleria dominicanensis*: (**A**)—protoconch, bar equals 50 µm; (**B**)—radula bar equals 50 µm; (**C**)—pigmentation of mantle along ctenidial lamellae, bar equals 500 µm.

Shell (Figure 6A–E) conic, moderately broad, with relatively low spire, up to 3.79 mm high, with about six whorls, spire height approximately 33% of shell height, and 66% of body whorl width. Teleoconch whorls slightly convex, evenly rounded, growing regularly in diameter. Aperture ovoid, moderately broad, with an angle at its upper part, outer lip simple, parietal lip complete, umbilicus slit-like. Teleoconch glossy, growth lines hardly visible, periostracum white to yellowish. Protoconch (Figure 7A) smooth, no sharp border between proto- and teleoconch. Operculum paucispiral, without any outgrowth. Measurements: Table 2.

Radula (Figure 7B) with central tooth with lateral angles highly expanded, ventral process well excavated, cusps rather long and massive, following the formula:

$$\frac{6-1-6}{1-1}$$

Median cusp twice longer than adjacent ones, basal cusps broad, located relatively high on lateral angles of central tooth. Lateral tooth with 3(4)–1–6 blunt cusps, inner marginal tooth with 22–24 sharp cusps, outer marginal tooth with 8–10 delicate sharp cusps.

Morphology and anatomy of soft parts

**Figure 8:**
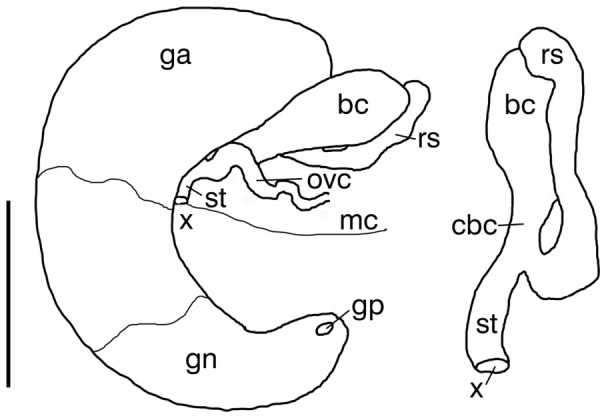
Female reproductive organs of *Hershleria dominicanensis* (bc—bursa copulatrix; cbc—bursal duct; ga—albumen gland; gn—capsule gland; gp—gonoporus; mc—posterior end of mantle cavity; ovc—coiled (renal) oviduct; rs—receptaculum seminis; st—sperm tube; x—second (copulatory) gonoporus); bar equals 1 mm.

**Figure 9:**
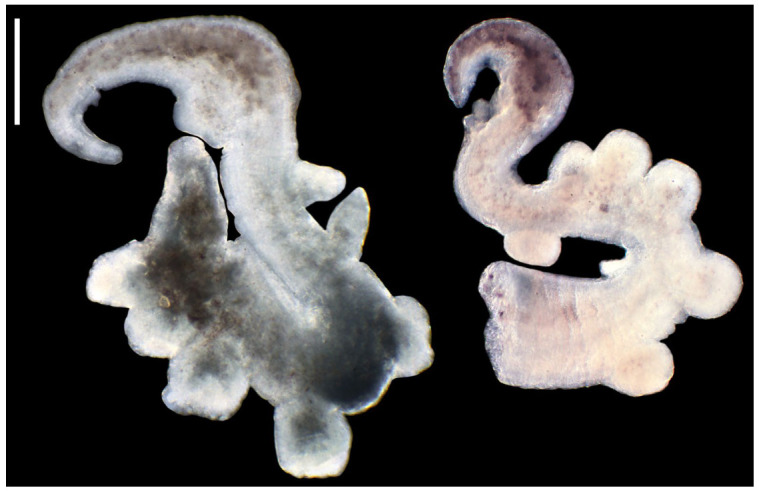
Penes of *Hershleria dominicanensis*; bar equals 200 µm.

The head pigmented black, eye spots present, hyperciliation of the tentacles. On the mantle some spots of pigment, lines of pigment along the bases of the ctenidial lamellae (Figure 7C), ctenidium with about 22 lamellae, osphradium small. Female reproductive organs typical of *Heleobia* [11,52,71,72] with big bursa copulatrix closely overlapping on albumen gland, with long duct, long and worm-shaped seminal receptacle with no distinct duct, sperm tube short, opening into posterior end of pallial cavity (Figure 8). Penis (Figure 9) long, flattened, tapering distally, with 5–6 stout, short-stalked apocrine glands arising from outer curvature at proximal part of penis. All glands similar in size. At the half of the penis length small and slim non-glandular outgrowth on the right side. On the left side, at base od terminal tapering section, flat outgrowth, terminal section with long spot of brown pigment.

**Differential diagnosis**

*Hershleria dominicanensis* differs from *Heleobia australis* in having a non-turreted shell with a narrower spire, less sharp basal cusps, and more cusps on cutting edge of the central tooth and lateral tooth of the radula (*H. australis crassa* M. Gaillard, 1974, while in *H. australis nana* Er. Marcus et Ev. Marcus, 1963, the radula is similar as *Hershleria dominicanensis*), smaller and flatter outgrowth at base of terminal tapering section of the penis, and five–six, instead of one, apocrine glands (anatomy of *H. australis*: [18]). *Heleobia australis* differs also in its brackish-water habitat (although there is a very low admixture of the sea water in the Dudu Cenote) and free-swimming larva [73]. In the case of the Dudu cenote, with a very limited access of the sea water, only direct development would be possible. Combined shell and penis characters distinguish *Hershleria dominicanensis* from all *Heleobia* whose anatomy is known (see Discussion for references). The molecular diagnostic for *COI* characters in comparison with all *Heleobia* sequences used in molecular analysis: binary: 11(A), 183(C), 282(C), 341(T); asymmetric: 9(A), 408(T), 510(G).

Geographically, but not molecularly, the closest cochliopid taxon is *Heleobops docimus* [3,11], recorded from the Dominican Republic. *Hershleria dominicanensis* differs from *Heleobops docimus* in shell morphology by having less flat whorls and an unreflexed outer lip, in radula’s central tooth with broader basal cusps and more cusps on its cutting edge, in a bigger bursa copulatrix, and in penis with much bigger apocrine glands, lack of a lobe close to the terminal end of the penis (in *Heleobops* present or not), presence of two non-glandular outgrowths, and brown pigmentation of the terminal part of the penis. From another cochliopid genus recorded from the Dominican Republic, *Pyrgophorus* [3,11], *Hershleria dominicanensis* differs in the radula by having one instead of two basal cusps, and in the penis by lacking a distal bifurcation and having less numerous but bigger papillae (apocrine glands).

**Distribution and habitat**

Known only from the type locality.

## 4. Discussion

Despite more than one hundred described species of *Heleobia* [74], anatomical characters have been described for only a few of them, the same concerns molecular data [24,51,52,59,63,75]. Considering all the descriptions of the anatomy of *Heleobia* [11,52,58,71,72,76,77,78,79,80,81], *Hershleria dominicanensis* differs in its penis, and molecularly is clearly distinct. Contrary to a species, a genus is not a natural unit whose threshold distinguishing value can be estimated. Thus, in a given systematic group, the values of genetic distances should be similar between the distinguished genera.

The geographic range of *Heleobia* covers South America, but only its southern part and a narrow belt along the eastern coast, expanding northward into Amazonia, and, on the eastern side of the Atlantic Ocean, in several areas around the Mediterranean and on the Atlantic coasts of South Morocco and the Netherlands [11,82]. However, as concerns the European *Heleobia*, Radoman [83] described the genus *Semisalsa* Radoman, 1974 for *S. dalmatica*, inhabiting the spring Pirovac on the Dalmatian coast of the Adriatic Sea, and representatives of *Semisalsa* were later found at many localities along the European coasts. Giusti and Pezzoli [84] created a distinct subfamily, Semisalsinae Giusti et Pezzoli, 1980. Later, *Semisalsa* was synonymized with *Eupaludestrina*. It should be noted that, according to Szarowska et al. [52], *E. dalmatica*, as well as all the other European brackish-water *Eupaludestrina*, should be synonymized with *E. stagnorum* (Gmelin, 1791) (described as *Helix stagnorum* Gmelin, 1791, from the Kaaskenswaters, Zierikzee, the Netherlands [85,86]. A species of *Eupaludestrina* is also known from the Canary Islands [87]. Our *COI* tree (Figure 3) does not support the monophyly of *Eupaludestrina,* as well as *Heleobia*, and *Heleobops* is not clearly distinct from the *Eupaludestrina*/*Heleobia* group. It has to be noted that the anatomy, as usual in the Truncatelloidea [88], is variable, and the anatomic characters of *Heleobops*, especially of its penis [3,11], may rather fall within the range observed for *Eupaludestrina*/*Heleobia*. Similarly, female reproductive organs showing no differences between *Hershleria* and *Heleobia* may not deny the genus distinctness of the former.

The assignment of *Heleobia australis* to the genus *Heleobia* was already questioned [53,59,81]. The occurrence of *Heleobia* in Hispaniola Island, thousands of kilometres far from all the other localities of this genus is improbable. Passive transport by birds, even over such long distances, cannot be excluded: such transportation was observed several times for the truncatelloid snails [88]. The Antilles are on the route of bird migration between S and N America [50]. South American *Heleobia charruana* (d’Orbigny, 1841) from Uruguay has been recorded from the United Kingdom (2003), the Netherlands (2014), and Belgium (2017) [57]. Despite the possibility of long-distance passive transport, considering the Cretaceous origin of Hispaniola, there was a long time for evolution of an invader to become molecularly distinct. One should expect the occurrence of *Hershleria* at the neighbouring islands, at least, but the minute truncatelloid fauna of the latter remains poorly studied so far. Fortunately, the Dudu cenote is a protected area, which supports the necessary protection of this taxon, so far unique to this small habitat. However, there is some human impact in this area visited by numerous tourists and divers; thus, monitoring of this snail seems important.

## 5. Conclusions

The snails inhabiting the Dudu cenote in the Dominican Republic belong to the family Cochliopidae and represent a new genus and species. This is confirmed by both morphological and molecular data. This finding enriches our knowledge of the still-poorly studied, but interesting, malacofauna of the Antilles.

## Figures and Tables

**Table 2:** Shell measurements (in mm): a—shell height; b—body whorl breadth; c—aperture height; d—spire height; e—aperture breadth; α—apex angle measured between the lines tangential to the spire; β—angle between the body whorl suture and the line perpendicular to the columella; M—mean; SD—standard deviation; Min—minimum value; Max—maximum value.

	a	b	c	d	e	α	β
Holotype	3.79	1.84	1.70	1.42	1.21	49	12
3K92	3.49	1.73	1.52	1.33	1.25	53	10
3K93	3.39	1.63	1.46	1.40	1.11	49	13
A1	3.42	1.68	1.55	1.29	1.09	51	13
A2	3.48	1.80	1.69	1.23	1.19	52	10
M	3.51	1.74	1.58	1.33	1.17	50.80	11.60
SD	0.16	0.09	0.11	0.08	0.07	1.79	1.52
Min	3.39	1.63	1.46	1.23	1.09	49	10
Max	3.79	1.84	1.70	1.42	1.25	53	13

## Data Availability

The sequences were deposited in GenBank (accession numbers: PZ349056-PZ349063, PZ349601, PZ349653-PZ349656) at https://www.ncbi.nlm.nih.gov (accessed on 20 April 2026).

## Supplementary Materials

The following supporting information can be downloaded at: https://www.mdpi.com/article/10.3390/ani16121810/s1. Supplementary File S1: Molecular Diagnostic Characters (MDCs) for *COI*.
