# Supplementary material for: A New Genus and Species of Cochliopidae Tryon, 1866 (Truncatelloidea) from the Dominican Republic
Source: Animals (Basel). 2026 Jun 11;16(12):1810. doi: 10.3390/ani16121810 (PMC13295324; doi:10.3390/ani16121810)
Supplement: Supplementary file 1 [file animals-16-01810-s001.zip › animals-4315207-supplementary.pdf]

Supplementary File S1: Molecular Diagnostic Characters (MDCs) for *COI*.

>3K92
GGACAACCAGAAACTTTACTTGGTGATGATCAGCTTTATAATGTTATTGTTACTGCGCAT
GCGTTTGTAATAATTTTTTTCTTAGTTATGCCTATAATAATTGGTGGATTTGGAAACTGA
TTAGTTCCTTTAATGTTAGGAGCTCCAGACATGGCTTTTCCTCGATTAAATAATATAAGT
TTCTGATTATTACCACCTGCTCTTCTTTTATTGCTTTCTTCAGCTGCAGTTGAAAGTGGT
GTGGGGACAGGATGAACTGTTTATCCTCCTCTGTCTGCAAACCTGGCTCACGCCGGTGGT
TCTGTAGATCTTGCAATTTTTTCTTTACATTTAGCGGGTGTTTCTTCTATTTTAGGTGCT
GTAAATTTTATTACTACAGTTATTAATATACGTTGAGGGGGTATGCCTCTTGAACGGCTT
TCTCTTTTTGTTTGATCAGTAAAGATTACAGCTATTTTACTTTTATTGTCTCTTCCTGTT
TTAGCTGGGGCTATTACTATACTTTTAACG
>3K93
GGACAACCAGAAACTTTACTTGGTGATGATCAGCTTTATAATGTTATTGTTACTGCACAT
GCGTTTGTAATAATTTTTTTCTTAGTTATGCCTATAATAATTGGTGGATTTGGAAACTGA
TTAGTTCCTTTAATGTTAGGAGCTCCAGACATGGCTTTTCCTCGATTAAATAATATAAGT
TTCTGATTATTACCACCTGCTCTTCTTTTATTGCTTTCTTCAGCTGCAGTTGAAAGTGGT
GTGGGGACAGGATGAACTGTTTATCCTCCTCTGTCTGCAAACCTGGCTCACGCCGGTGGT
TCTGTAGATCTTGCAATTTTTTCTTTACATTTAGCGGGTGTTTCTTCTATTTTAGGTGCT
GTAAATTTTATTACTACAGTTATTAATATACGTTGAGGGGGTATGCCTCTTGAACGGCTT
CCTCTTTTTGTTTGATCAGTAAAGATTACAGCTATTTTACTTTTATTGTCTCTTCCTGTT
TTAGCTGGGGCTATTACTATACTTTTAACG
>3M24
GGACAACCAGAAACTTTACTTGGTGATGATCAGCTTTATAATGTTATTGTTACTGCACAT
GCGTTTGTAATAATTTTTTTCTTAGTTATGCCTATAATAATTGGTGGATTTGGAAACTGA
TTAGTTCCTTTAATGTTAGGAGCTCCAGACATGGCTTTTCCTCGATTAAATAATATAAGT
TTCTGATTATTACCACCTGCTCTTCTTTTATTGCTTTCTTCAGCTGCAGTTGAAAGTGGT
GTGGGGACAGGATGAACTGTTTATCCTCCTCTGTCTGCAAACCTGGCTCACGCCGGTGGT
TCTGTAGATCTTGCAATTTTTTCTTTACATTTAGCGGGTGTTTCTTCTATTTTAGGTGCT
GTAAATTTTATTACTACAGTTATTAATATACGTTGAGGGGGTATGCCTCTTGAACGGCTT
CCTCTTTTTGTTTGATCAGTAAAGATTACAGCTATTTTACTTTTATTGTCTCTTCCTGTT
TTAGCTGGGGCTATTACTATACTTTTAACG
>3M25
GGACAACCAGAAACTTTACTTGGTGATGATCAGCTTTATAATGTTATTGTTACTGCACAT
GCGTTTGTAATAATTTTTTTCTTAGTTATGCCTATAATAATTGGTGGATTTGGAAACTGA
TTAGTTCCTTTAATGTTAGGAGCTCCAGACATGGCTTTTCCTCGATTAAATAATATAAGT
TTCTGATTATTACCACCTGCTCTTCTTTTATTGCTTTCTTCAGCTGCAGTTGAAAGTGGT
GTGGGGACAGGATGAACTGTTTATCCTCCTCTGTCTGCAAACCTGGCTCACGCCGGTGGT
TCTGTAGATCTTGCAATTTTTTCTTTACATTTAGCGGGTGTTTCTTCTATTTTAGGTGCT
GTAAATTTTATTACTACAGTTATTAATATACGTTGAGGGGGTATGCCTCTTGAACGGCTT
CCTCTTTTTGTTTGATCAGTAAAGATTACAGCTATTTTACTTTTATTGTCTCTTCCTGTT
TTAGCTGGGGCTATTACTATACTTTTAACG
>MT295136
GGACAGCCTGGAACTTTACTTGGTGACGATCAGCTTTATAATGTTATTGTTACTGCGCAT
GCGTTTGTAATAATTTTTTTCTTAGTTATGCCTATGATGATTGGTGGGTTTGGAAATTGA
TTAGTTCCTTTAATGCTGGGAGCTCCAGACATGGCTTTTCCTTACTTAAATAATATAAGT
TTTTGATTATTACCACCTGCTCTTCTTTTATTACTTTCTTCAGCTGCAGTTGAAAGTGGT
GTAGGAACAGGATGAACTGTTTATCCGCCTTTATCTGCAAATCTGGCTCATGCTGGTGGT

TCTGTAGATCTTGCAATTTTTTCTTTACACTTAGCAGGTGCTTCTTCTATTTTAGGTGCT
GTAAATTTTATTACTACAGTTATTAATATACGTTGAGGGGGTATGCCACTTGAACGGCTT
CCTCTTTTTGTCTGATCAGTAAAGATTACAGCTATTTTACTTCTATTGTCTCTTCCTGTT
TTAGCTGGAGCTATTACTATGCTTTTAACA
>JQ972708
GGACAGCCTGGAACTTTACTTGGTGACGATCAGCTTTATAATGTTATTGTTACTGCGCAT
GCGTTTGTAATAATTTTTTTCTTAGTTATGCCTATGATGATTGGTGGGTTTGGAAATTGA
TTAGTTCCTTTAATGCTAGGGGCTCCAGACATGGCTTTTCCTCGATTAAATAATATAAGT
TTTTGATTATTACCACCTGCTCTTCTTTTATTACTTTCTTCAGCTGCAGTTGAAAGTGGT
GTAGGGACAGGATGAACTGTTTATCCGCCTTTATCTGCAAATCTGGCTCATGCCGGTGGT
TCTGTAGATCTTGCAATTTTCTCTTTACACTTAGCGGGTGCTTCTTCTATTTTAGGTGCT
GTAAATTTTATTACTACAGTTATTAATATACGTTGAGGGGGTATGCCACTTGAACGGCTT
CCTCTTTTTGTCTGATCAGTAAAGATTACAGCTATTTTACTTTTATTGTCTCTTCCTGTT
TTAGCTGGAGCTATTACTATGCTTTTAACA
>EU938132
GGACAACCTGGAACTTTACTTGGTGACGATCAGCTTTATAATGTTATTGTTACTGCACAT
GCGTTTGTAATAATTTTTTTCTTAGTTATGCCCATGATGATTGGCGGGTTTGGAAATTGA
TTAGTTCCTTTAATATTAGGAGCTCCAGACATAGCTTTTCCTCGATTAAATAATATAAGT
TTTTGATTGTTACCACCTGCTCTTCTTTTATTACTCTCTTCAGCTGCAGTTGAAAGTGGT
GTAGGAACAGGATGGACTGTTTATCCTCCTCTATCTGGAAATCTAGCCCATGCTGGTGGT
TCTGTAGATCTTGCAATTTTTTCTCTACATTTAGCAGGTGCTTCTTCTATTTTAGGTGCT
GTAAATTTCATTACTACAGTTATTAATATGCGTTGAGGAGGTATGCCACTTGAACGACTT
CCTCTCTTTGTCTGATCAGTAAAAATTACAGCCATTTTACTTTTATTATCCCTTCCTGTT
TTGGCTGGAGCTATTACTATACTTCTAACA
>JQ973024
GGACAACCTGGAACTTTGCTTGGTGACGATCAGCTTTATAATGTTATTGTTACTGCACAT
GCGTTTGTAATAATTTTTTTCTTAGTTATGCCTATAATAATTGGTGGGTTTGGAAACTGA
TTAGTTCCTTTAATATTAGGAGCTCCAGACATAGCTTTTCCTCGATTAAATAATATAAGT
TTTTGATTATTACCACCTGCTCTTCTTTTATTACTTTCTTCAGCTGCAGTTGAGAGCGGT
GTAGGGACGGGATGAACTGTTTATCCCCCTCTATCTGCAAATTTAGCTCACGCTGGTGGT
TCTGTAGATCTTGCAATTTTTTCTTTACACTTAGCGGGTGCTTCTTCTATTTTAGGTGCT
GTAAATTTTATTACTACAGTTATTAACATACGCTGAGGAGGTATGCCGCTTGAACGGCTT
CCTCTCTTTGTTTGATCAGTAAAGATTACAGCTATTTTACTTTTATTGTCCCTTCCTGTT
TTAGCTGGAGCCATTACTATACTTTTAACA
>JQ973025
GGACAACCTGGAACTTTGCTTGGTGACGATCAGCTTTATAATGTTATTGTTACTGCACAT
GCGTTTGTAATAATTTTTTTCTTAGTTATGCCTATAATAATTGGTGGGTTTGGAAACTGA
TTAGTTCCTTTAATATTAGGAGCTCCAGACATAGCTTTTCCTCGATTAAATAATATAAGT
TTTTGATTGTTACCACCTGCTCTTCTTTTATTACTTTCTTCAGCTGCAGTTGAGAGCGGT
GTAGGGACGGGATGAACTGTTTATCCCCCTCTATCTGCAAATTTAGCTCACGCTGGTGGT
TCTGTAGATCTTGCAATTTTTTCTTTACACTTAGCGGGTGCTTCTTCTATTTTAGGTGCT
GTAAATTTTATTACTACAGTTATTAACATACGCTGAGGAGGTATGCCGCTTGAACGGCTT
CCTCTCTTTGTTTGATCAGTAAAGATTACAGCTATTTTACTTTTATTGTCCCTTCCTGTT
TTAGCTGGAGCCATTACTATACTTTTAACA
>KM213723
GGACAACCTGGAACTTTGCTTGGTGACGATCAGCTTTATAATGTTATTGTTACTGCACAT
GCGTTTGTAATAATTTTTTTCTTAGTTATGCCTATAATAATTGGCGGGTTTGGAAACTGA
TTAGTTCCTTTAATATTAGGAGCTCCAGACATAGCTTTTCCTCGATTAAATAATATAAGT
TTTTGATTGTTGCCACCTGCTCTTCTTTTATTACTTTCTTCAGCTGCAGTTGAGAGCGGT
GTAGGGACGGGATGAACTGTTTATCCCCCTCTATCTGCAAATTTAGCTCACGCTGGTGGT
TCTGTAGATCTTGCAATTTTTTCTTTACACTTAGCAGGTGCTTCTTCTATTTTAGGTGCT

GTAAATTTTATTACTACAGTTATTAACATACGCTGAGGAGGTATGCCGCTTGAACGGCTT
CCTCTCTTTGTTTGATCAGTAAAGATTACAGCTATTTTACTTTTATTGTCCCTTCCTGTT
TTAGCTGGAGCCATTACTATACTCTTAACA
>AF213347
GGACAACCCGGGACTTTACTTGGTGACGATCAGCTTTATAATGTTATCGTTACTGCGCAT
GCGTTTGTAATAATTTTTTTCTTAGTTATGCCCATAATAATTGGTGGGTTTGGAAATTGA
TTAGTTCCTTTAATATTAGGGGCTCCAGACATAGCTTTTCCTCGGTTAAATAATATAAGT
TTTTGATTACTACCACCTGCTCTTCTTTTATTACTTTCTTCAGCTGCAGTTGAGAGCGGT
GTAGGTACAGGATGAACTGTTTATCCTCCTTTATCTGCAAATCTAGCTCATGCTGGGGGT
TCTGTAGATCTTGCGATTTTTTCTTTACACTTAGCAGGTGCTTCTTCTATTTTAGGTGCT
GTAAATTTTATTACTACAGTTATTAATATACGTTGAGGAGGTATGCCACTTGAACGGCTT
CCTCTCTTTGTTTGATCAGTAAAGATTACGGCTATTTTACTTTTATTATCTCTTCCTGTT
TTAGCTGGAGCTATTACTATGCTTTTAACT
>MN921143
GGACAACCCGGAACTTTACTTGGTGACGATCAGCTTTATAATGTTATTGTTACTGCGCAT
GCGTTTGTAATAATTTTTTTCTTAGTTATGCCTATAATAATTGGTGGGTTTGGGAATTGA
TTAATTCCTTTAATATTAGGAGCTCCAGATATAGCTTTTCCTCGATTAAATAATATGAGT
TTTTGGTTGTTACCACCTGCTCTTCTTTTATTACTTTCTTCAGCTGCAGTTGAAAGTGGT
GTGGGGACAGGGTGAACTGTTTATCCTCCTTTATCTGCAAATCTGGCTCATGCTGGTGGT
TCTGTAGATCTTGCAATTTTTTCTTTGCATTTAGCAGGTGCTTCTTCTATTTTAGGTGCT
GTAAATTTTATTACTACAGTTATTAATATACGCTGAGGGGGTATGCCACTTGAACGGCTT
CCTCTTTTTGTTTGATCAGTAAAGATTACAGCTATTTTACTTTTATTATCCCTTCCTGTT
TTAGCTGGAGCTATTACTATACTTTTAACA
>JQ973045
GGACAACCCGGAACTTTACTTGGTGACGATCAGCTTTATAATGTTATTGTTACTGCGCAT
GCGTTTGTAATAATTTTTTTCTTAGTTATGCCCATGATGATTGGCGGGTTTGGAAATTGA
TTAGTTCCTTTAATATTAGGGGCTCCAGACATAGCTTTTCCTCGATTAAATAACATGAGT
TTTTGATTGTTACCACCTGCTCTTCTTTTATTACTTTCTTCAGCTGCAGTTGAAAGTGGT
GTAGGGACGGGATGAACTGTTTATCCTCCTCTATCTGCAAATCTGGCTCACGCTGGTGGT
TCTGTAGATCTTGCAATTTTTTCTTTACATCTAGCAGGTGCTTCTTCTATTTTAGGTGCT
GTAAATTTTATTACTACAGTTATTAACATACGTTGACGAGGTATACCACTTGAGCGGCTT
CCTCTCTTTGTCTGATCAGTAAAGATTACAGCTATTTTACTTTTATTATCCCTTCCTGTT
TTAGCTGGAGCTATTACTATACTTTTAACA
>KF658072
GGACAACCCGGAACTTTACTTGGTGACGATCAGCTTTATAATGTTATTGTTACTGCGCAT
GCGTTTGTAATAATTTTTTTCTTAGTTATGCCCATGATGATTGGCGGGTTTGGAAATTGA
TTAGTTCCTTTAATATTAGGGGCTCCAGACATAGCTTTTCCTCGATTAAATAACATGAGT
TTTTGATTGTTACCACCTGCTCTTCTTTTATTACTTTCTTCAGCTGCAGTTGAAAGTGGT
GTAGGGACGGGATGAACTGTTTATCCTCCTCTATCTGCAAATCTGGCTCACGCTGGTGGT
TCTGTAGATCTTGCAATTTTTTCTTTACATCTAGCAGGTGCTTCTTCTATTTTAGGTGCT
GTAAATTTTATTACTACAGTTATTAACATACGTTGACGAGGTATACCACTTGAGCGGCTT
CCTCTCTTTGTCTGATCAGTAAAGATTACAGCTATTTTACTTTTATTATCCCTTCCTGTT
TTAGCTGGAGCTATTACTATACTTTTAACA
>KR816827
GGACAACCCGGAACTTTACTTGGTGACGATCAGCTTTATAATGTTATTGTTACTGCGCAT
GCGTTTGTAATAATTTTTTTCTTAGTTATGCCCATAATGATTGGCGGGTTTGGAAATTGA
TTAGTTCCTTTAATATTAGGGGCTCCAGACATAGCTTTTCCTCGATTAAACAACATGAGT
TTTTGATTGTTACCACCTGCTCTTCTTTTATTACTTTCTTCAGCTGCAGTTGAAAGTGGT
GTAGGGACGGGATGAACTGTTTATCCTCCTCTATCTGCAAATTTGGCTCACGCTGGTGGT
TCTGTAGATCTTGCAATTTTTTCTTTACATCTAGCGGGTGCTTCTTCTATTTTAGGTGCT
GTAAATTTTATTACTACAGTTATTAACATACGTTGACGGGGTATACCGCTTGAGCGGCTT

CCTCTCTTTGTCTGATCAGTAAAGATTACAGCTATTTTACTTTTATTATCCCTTCCTGTT
TTAGCTGGAGCTATTACTATACTTTTAACA
>JQ973050
GGACAACCCGGAACTTTACTTGGTGACGATCAGCTTTATAACGTTATTGTTACTGCGCAT
GCGTTTGTAATAATTTTTTTCTTAGTTATGCCTATGATGATTGGCGGGTTTGGAAATTGA
TTAGTTCCTTTAATATTAGGAGCTCCAGACATAGCTTTTCCTCGATTAAATAACATGAGT
TTTTGATTGTTACCACCTGCTCTTCTTTTATTACTTTCTTCAGCTGCAGTTGAAAGTGGT
GTAGGGACGGGATGAACTGTTTATCCTCCTCTATCTGCAAATCTGGCTCACGCCGGTGGT
TCTGTAGATCTTGCAATTTTTTCTTTACATCTAGCGGGTGCTTCTTCTATTTTAGGTGCT
GTAAATTTTATTACTACAGTTATTAACATACGTTGACGGGGTATACCACTTGAGCGGCTT
CCTCTCTTTGTCTGATCAGTGAAGATTACAGCTATTTTACTTTTATTATCCCTTCCTGTT
TTAGCTGGAGCTATTACTATGCTTTTAACA
>KF658185
GGACAACCCGGAACTTTACTTGGTGACGATCAGCTTTATAATGTTATTGTTACTGCCCAT
GCATTTGTAATAATTTTTTTCTTAGTCATGCCTATGATGATTGGCGGGTTTGGAAATTGA
TTAATTCCTTTAATATTAGGAGCTCCAGACATAGCTTTTCCTCGATTAAATAACATGAGT
TTTTGATTGTTACCACCTTCTCTTCTTTTATTACTTTCTTCAGCTGCAGTTGAAAGTGGT
GTAGGGACGGGATGAACTGTTTATCCTCCTCTATCTGCAAATCTGGCCCACGCCGGTGGT
TCTGTAGATCTTGCAATTTTTTCTTTACATCTAGCGGGTGCTTCTTCTATTTTAGGTGCT
GTAAATTTTATTACTACAGTTATTAACATACGTTGACGTGGTATGCCACTTGAGCGGCTT
CCTCTCTTTGTCTGATCAGTAAAGGTTACAGCTATTTTACTTTTATTATCCCTTCCTGTT
TTAGCTGGAGCTATTACTATACTTTTAACA
>JQ973052
GGACAACCCGGAACTTTACTTGGTGACGATCAGCTTTATAATGTTATTGTTACTGCGCAT
GCGTTTGTAATAATTTTTTTCTTAGTTATGCCTATGATGATTGGTGGGTTTGGAAATTGA
TTAGTTCCTTTAATATTAGGAGCTCCAGACATAGCTTTTCCTCGATTAAATAACATGAGT
TTTTGATTGTTACCACCTGCTCTTCTTTTATTACTTTCTTCAGCTGCAGTTGAAAGTGGT
GTAGGGACGGGATGAACTGTTTATCCTCCTCTATCTGCAAATCTGGCTCACGCTGGTGGT
TCTGTAGATCTTGCAATTTTTTCTTTACATCTAGCGGGTGCTTCTTCTATTTTAGGTGCT
GTAAATTTTATTACTACAGTTATTAACATACGTTGACGGGGTATACCACTTGAGCGGCTT
CCTCTATTTGTTTGATCAGTAAAGATTACAGCTATTTTACTTTTATTATCCCTTCCTGTT
TTAGCTGGAGCTATTACTATACTTTTAACA
>KF658150
GGACAACCCGGAACTTTACTTGGTGACGATCAGCTTTATAATGTTATTGTTACTGCGCAT
GCGTTTGTAATAATTTTTTTCTTAGTTATACCTATGATAATTGGTGGGTTTGGAAATTGA
TTAGTTCCTTTAATATTAGGAGCTCCAGACATAGCTTTTCCTCGATTAAATAACATAAGT
TTTTGATTGTTACCACCTGCTCTTCTTTTATTACTTTCTTCAGCTGCAGTTGAAAGTGGT
GCAGGGACGGGATGAACTGTCTATCCTCCTCTATCTGCAAATCTGGCTCACGCTGGTGGT
TCTGTAGATCTTGCAATTTTTTCTTTGCATCTAGCGGGTGCTTCTTCTATTTTAGGTGCT
GTAAATTTTATTACTACAGTTATTAACATACGTTGACGGGGTATACCACTTGAGCGGCTT
CCTCTCTTTGTCTGATCAGTAAAGATTACAGCTATTTTACTTTTATTATCCCTTCCTGTT
TTAGCTGGAGCTATTACTATACTTTTAACA
>MN094525
GGACAACCCGGAACTTTACTTGGTGACGATCAGCTTTATAATGTTATTGTTACTGCGCAT
GCGTTTGTAATAATTTTTTTCTTAGTTATACCTATGATGATTGGCGGGTTTGGAAATTGA
TTAGTTCCTTTAATATTAGGAGCTCCAGACATAGCTTTTCCTCGATTAAATAACATGAGT
TTTTGATTGTTACCGCCTGCCCTTCTCTTATTACTTTCTTCAGCTGCAGTTGAAAGTGGT
GTAGGGACGGGATGAACTGTTTATCCTCCTCTATCTGCAAATCTGGCTCACGCTGGTGGT
TCTGTAGATCTTGCAATTTTTTCTTTACATCTAGCGGGTGCTTCTTCTATTTTAGGTGCT
GTAAATTTTATTACTACAGTTATTAACATACGTTGACGGGGTATACCACTTGAGCGGCTT
CCTCTCTTTGTCTGATCAGTAAAGATTACAGCTATTTTACTTTTATTATCCCTTCCTGTT

TTAGCTGGAGCTATTACTATACTTTTAACA
>MN094494
GGACAACCCGGAACTTTACTTGGTGACGATCAGCTTTACAATGTTATTGTTACTGCGCAT
GCGTTTGTAATAATTTTTTTCTTAGTTATGCCTATGATGATTGGCGGGTTTGGAAATTGA
TTAGTTCCTTTAATATTAGGAGCTCCAGACATAGCTTTTCCTCGATTAAATAATATGAGT
TTTTGATTGTTACCGCCTGCTCTTCTTTTATTACTTTCTTCAGCTGCAGTTGAAAGTGGT
GTAGGGACGGGATGAACTGTTTATCCTCCTCTATCTGCAAATCTGGCTCACGCTGGTGGT
TCTGTAGATCTTGCAATTTTTTCTTTACATCTAGCGGGTGCTTCTTCTATTTTAGGTGCT
GTAAATTTTATCACTACAGTTATTAACATACGTTGACGGGGTATGCCACTTGAGCGGCTT
CCTCTCTTTGTCTGATCAGTAAAGATTACAGCCATTTTACTTTTATTATCCCTTCCTGTT
TTAGCTGGAGCTATTACTATACTTTTAACA
>MN094522
GGACAACCCGGAACTTTACTTGGTGACGATCAGCTTTATAATGTTATTGTTACTGCGCAT
GCGTTTGTAATAATTTTTTTCTTAGTTATGCCTATGATGATTGGCGGGTTTGGAAATTGA
TTAGTTCCTTTAATATTAGGAGCTCCAGACATAGCTTTTCCTCGATTAAATAACATGAGT
TTTTGATTGTTACCGCCTTCTCTTCTTTTATTACTTTCTTCAGCTGCAGTTGAAAGCGGT
GTAGGGACGGGATGAACTGTTTATCCTCCCCTGTCTGCAAATCTGGCTCACGCTGGTGGT
TCTGTAGATCTTGCAATTTTTTCTTTACACCTAGCGGGTGCTTCTTCTATTTTAGGTGCT
GTAAATTTTATCACTACAGTTATCAACATACGTTGAGGGGGTATGCCACTTGAGCGGCTT
CCTCTTTTTGTCTGATCAGTAAAAATTACAGCTATTTTACTTTTATTATCCCTTCCTGTT
TTAGCTGGAGCTATTACTATACTTTTAACA
>MN094463
GGACAACCTGGAACTTTACTTGGTGACGATCAGCTTTATAATGTTATTGTTACTGCGCAT
GCGTTTGTAATAATTTTTTTCTTAGTTATGCCTATGATGATTGGTGGGTTTGGAAATTGA
TTAGTTCCTTTAATATTAGGAGCTCCAGACATAGCTTTTCCTCGATTAAATAACATGAGT
TTTTGATTGTTACCGCCTGCTCTTCTTTTATTACTTTCTTCAGCTGCAGTTGAAAGTGGT
GTAGGGACGGGATGAACTGTTTATCCTCCTCTATCTGCAAATCTGGCTCACGCTGGTGGT
TCTGTAGATCTTGCAATTTTTTCTTTACATCTAGCAGGTGCTTCTTCTATTTTAGGTGCT
GTAAATTTTATTACCACAGTTATTAACATACGTTGACGGGGTATGCCACTTGAGCGGCTT
CCTCTCTTTGTCTGATCAGTAAAGATTACAGCTATTTTACTTTTATTATCCCTTCCTGTT
TTAGCTGGAGCTATTACTATACTTTTAACA
>OP630467
GGACAACCCGGAACTTTGCTTGGTGATGATCAGCTTTATAATGTTATTGTTACTGCGCAT
GCGTTTGTAATAATTTTTTTCTTAGTTATGCCTATGATGATTGGTGGGTTTGGAAATTGA
CTAGTTCCTTTAATATTAGGAGCTCCAGACATAGCTTTTCCTCGATTAAATAACATAAGT
TTTTGATTGTTACCGCCTGCTCTTCTTTTATTACTTTCTTCAGCTGCAGTTGAAAGTGGT
GTAGGGACAGGATGAACTGTTTATCCTCCTCTATCTGCAAATCTGGCTCATGCTGGTGGT
TCCGTAGATCTTGCAATTTTTTCTTTACATTTAGCAGGTGCTTCTTCTATTTTAGGTGCT
GTAAATTTTATTACTACAGTTATTAACATACGTTGAGGGGGTATGCCGCTTGAACGGCTT
CCTCTTTTTGTCTGATCAGTAAAGATTACAGCTATTTTACTTTTATTATCCCTTCCTGTT
TTAGCTGGAGCTATTACTATACTTTTAACA
>MF447993
GGACAACCCGGAACTTTGCTTGGTGACGATCAGCTTTATAATGTTATTGTTACTGCGCAT
GCGTTTGTAATAATTTTTTTTTTAGTTATGCCTATAATAATTGGCGGGTTTGGGAATTGA
TTAATTCCTTTAATATTAGGAGCTCCAGACATAGCTTTTCCCCGATTAAATAACATAAGT
TTTTGATTATTACCGCCTGCTCTTCTATTATTACTCTCTTCAGCTGCAGTTGAAAGTGGG
GTAGGGACAGGATGAACTGTTTATCCTCCTCTATCTGCAAATCTTGCTCATGCTGGTGGT
TCTGTAGATCTTGCAATTTTTTCTTTACATTTAGCAGGTGCTTCTTCTATTTTAGGTGCT
GTAAATTTTATTACTACAATTATTAATATACGTTGAGGGGGTATGCCACTTGAACGGCTT
CCTCTTTTTGTCTGATCAGTAAAGATTACAGCTATTTTACTTTTATTGTCCCTCCCTGTT
TTAGCTGGAGCTATTACTATGCTTTTAACA

>OQ847325
GGACAACCTGGAACTTTACTTGGTGACGATCAGCTTTATAATGTTATTGTTACTGCGCAT
GCGTTTGTAATAATTTTTTTTTAGTTATGCCTATGATAATTGGCGGGTTTGGGAATTGA
TTAATTCCTTTAATATTGGGAGCTCCAGACATAGCTTTTCCCCGATTAAATAACATAAGT
TTTTGATTATTACCTCCTGCTCTTTTATTATTACTCTCTTCAGCTGCAGTTGAAAGTGGG
GTAGGGACAGGATGAACTGTCTATCCTCCTCTATCTGCAAATCTTGCTCATGCTGGTGGT
TCTGTAGATCTTGCAATTTTTTCTTTACATTTAGCAGGTGCTTCTTCTATTTTAGGTGCT
GTAAATTTTATTACTACAGTTATTAATATACGTTGAGGGGGTATACCACTTGAACGGCTT
CCTCTTTTTGTCTGATCAGTAAAGATTACAGCTATTTTACTTTTATTGTCCCTCCCTGTT
TTAGCTGGAGCTATTACTATGCTTTTAACA
>JQ973043
GGACAACCCGGAACTTTACTTGGTGACGATCAGCTTTATAATGTTATTGTAACTGCGCAT
GCGTTTGTAATAATTTTTTTCTTAGTTATACCTATAATGATTGGTGGCTTTGGAAATTGA
CTGGTTCCTTTAATATTAGGAGCTCCAGACATAGCTTTTCCTCGATTAAATAACATAAGT
TTTTGATTGTTACCACCTGCCCTTCTTTTATTACTTTCTTCAGCTGCAGTTGAGAGTGGT
GTGGGGACAGGATGAACTGTTTATCCTCCTCTATCTGCAAATCTGGCTCATGCTGGTGGT
TCTGTAGATCTTGCAATTTTTTCTTTACATTTAGCAGGTGCTTCTTCTATTTTAGGTGCT
GTAAATTTTATTACTACAGTTATTAATATACGTTGAGGGGGTATACCACTCGAACGGCTT
CCTCTTTTTGTTTGATCAGTAAAGATTACAGCTATTTTACTTTTATTGTCTCTTCCTGTT
TTAGCTGGAGCTATTACTATACTCTTAACA
>MH729608
GGACAACCCGGAACTTTACTTGGTGACGATCAGCTTTATAATGTTATTGTAACTGCGCAT
GCGTTTGTAATAATTTTTTTTTAGTTATACCTATGATGATTGGTGGCTTTGGAAATTGA
CTGGTTCCTTTAATATTAGGAGCTCCAGACATAGCTTTTCCTCGATTAAATAACATAAGT
TTTTGATTGTTACCACCTGCCCTTCTTTTATTACTTTCTTCAGCTGCAGTTGAGAGTGGT
GTGGGGACAGGATGAACTGTTTACCCTCCTCTATCTGCAAATCTGGCTCATGCTGGTGGT
TCTGTAGATCTTGCAATTTTTCTTTACATTTAGCAGGTGCTTCTTCTATTTTAGGTGCT
GTAAATTTTATTACTACAGTTATTAATATACGTTGAGGGGGTATGCCACTCGAACGGCTT
CCTCTTTTTGTTTGATCAGTAAAAATTACAGCTATTTTACTTTTATTGTCTCTTCCTGTT
TTAGCTGGAGCTATTACTATACTTTTAACA
>MN921148
GGACAACCCGGAACTTTACTTGGTGACGATCAGCTTTATAATGTTATTGTTACTGCGCAT
GCGTTTGTAATAATTTTTTTCTTAGTTATACCTATAATGATTGGTGGCTTTGGAAATTGA
CTGATTCCTTTAATATTAAGAGCTCCAGACATAGCTTTTCCTCGATTAAATAACATAAGT
TTTTGATTGTTACCGCCTGCCCTTCTTTTACTACTTTCTTCAGCTGCAGTTGAGAGTGGT
GTGGGGACAGGATGAACTGTTTATCCTCCTTTATCTGCAAATCTGGCTCATGCTGGTGGT
TCTGTAGATCTTGCAATCTTTTCTTTACATTTAGCAGGTGCTTCTTCTATTTTAGGTGCT
GTAAATTTTATTACTACAGTTATTAATATACGTTGAGGGGGTATGCCACTCGAACGGCTT
CCTCTTTTTGTTTGATCAGTAAAGATTACAGCTATTTTACTTTTATTGTCCCTTCCTGTT
TTAGCTGGAGCTATTACTATACTTTTAACA
>KF658141
GGACAACCCGGAACTTTACTTGGTGACGATCAGCTTTATAATGTTATTGTTACTGCGCAT
GCGTTTGTAATAATTTTTTTCTTAGTTATACCTATGATGATTGGTGGCTTTGGAAATTGA
CTAATTCCTTTAATATTAGGAGCTCCAGACATAGCTTTTCCTCGATTAAATAACATAAGT
TTTTGATTGTTACCGCCTGCCCTTCTTTTACTACTTTCTTCAGCTGCAGTTGAGAGTGGT
GTGGGGACAGGATGAACTGTTTATCCTCCTTTATCTGCAAATCTGGCTCATGCTGGTGGT
TCTGTAGATCTTGCAATCTTTTCTTTACATTTAGCAGGTGCTTCTTCTATTTTAGGTGCT
GTAAATTTTATTACTACAGTTATTAATATACGTTGAGGGGGTATGCCACTCGAACGGCTT
CCTCTTTTTGTTTGATCAGTAAAGATTACAGCTATTTTACTTTTATTGTCCCTTCCTGTT
TTAGCTGGAGCTATTACTATACTTTTAACA
>JQ972709

GGACAACCCGGAACTTTACTTGGTGACGATCAGCTTTATAATGTTATTGTCACTGCGCAT
GCGTTTGTAATAATTTTTTTCTTAGTTATGCCTATGATGATTGGTGGGTTTGGAAATTGA
CTAGTTCCTTTAATATTAGGAGCTCCAGACATAGCTTTTCCTCGACTAAATAATATGAGT
TTTTGATTGCTACCGCCTGCTCTTCTTTTATTACTTTCTTCAGCTGCAGTTGAGAGTGGT
GTGGGGACAGGATGAACTGTTTATCCTCCTTTATCTGCAAATCTGGCTCATGCTGGTGGT
TCTGTAGATCTTGCAATTTTTTCTTTACATTTAGCAGGTGCTTCTTCTATTTTAGGTGCT
GTAAATTTTATTACTACAGTTATTAATATACGTTGAGGGGGTATGCCACTCGAACGGCTT
CCTCTTTTTGTCTGATCAGTAAAAATTACAGCTATTTTACTTTTATTGTCCCTTCCTGTT
TTAGCTGGAGCTATTACCATACTCTTAACA
>KM220907
GGACAACCCGGAACTTTACTTGGTGACGATCAGCTTTATAATGTTATTGTCACTGCGCAT
GCGTTTGTAATAATTTTTTTCTTAGTTATGCCTATGATGATTGGTGGGTTTGGAAATTGA
CTAGTTCCTTTAATATTAGGAGCTCCAGACATAGCTTTTCCTCGACTAAATAATATGAGT
TTTTGGCTGTTACCGCCTGCTCTTCTTTTATTACTTTCTTCAGCTGCAGTTGAGAGTGGT
GTGGGGACAGGATGAACTGTTTATCCTCCTTTATCTGCAAATCTGGCTCATGCTGGTGGT
TCTGTAGATCTTGCAATTTTTTCTTTACATTTAGCAGGTGCTTCTTCTATTTTAGGTGCT
GTAAATTTTATTACTACAGTTATTAATATACGTTGAGGGGGTATGCCACTCGAACGGCTT
CCTCTTTTTGTCTGATCAGTAAAAATTACAGCTATTTTACTTTTATTGTCTCTTCCTGTT
TTAGCTGGAGCTATTACCATACTCTTAACA
>MW717674
GGACAACCCGGAACTTTACTTGGTGACGATCAGCTTTATAATGTTATTGTTACTGCGCAT
GCGTTTGTAATAATTTTTTTCTTAGTTATGCCTATGATAATTGGTGGATTTGGAAACTGA
CTAGTTCCTTTAATATTAGGAGCTCCAGACATAGCTTTTCCTCGGTTAAATAATATGAGT
TTTTGACTGTTACCGCCTGCTCTTCTTTTATTACTTTCTTCAGCTGCAGTTGAGAGAGGT
GTGGGAACAGGATGAACTGTTTATCCCCCTTTATCTGCAAATCTGGCCCATGCTGGTGGT
TCTGTAGATCTTGCAATTTTTTCTTTACATTTAGCAGGTGCTTCTTCTATTTTAGGTGCT
GTAAACTTTATTACTACAGTTATTAATATACGTTGAGGGGGTATGCCACTCGAACGGCTT
CCTCTCTTTGTTTGATCAGTAAAGATTACGGCTATCTTACTTTTATTGTCCCTTCCTGTT
TTAGCTGGGGCTATTACCATACTCTTAACA
>KM220904
GGACAACCCGGAACTTTACTTGGTGACGATCAGCTTTATAATGTTATTGTTACTGCGCAT
GCGTTTGTAATAATTTTTTTCTTAGTTATGCCTATAATGATTGGCGGGTTTGGAAATTGA
CTAGTTCCTTTAATATTAGGAGCTCCAGACATAGCTTTTCCTCGGTTAAATAATATGAGT
TTTTGACTGTTACCGCCTGCTCTTCTTTTATTACTTTCTTCAGCTGCAGTTGAGAGTGGT
GTGGGAACAGGATGAACTGTTTATCCTCCTTTATCTGCAAATCTGGCTCATGCTGGTGGT
TCTGTAGATCTTGCAATTTTTTCTTTACATTTAGCAGGTGCTTCTTCTATTTTAGGTGCT
GTAAACTTTATTACTACAGTTATTAACATACGTTGAGGGGGTATGCCACTCGAACGGCTT
CCTCTTTTTGTTTGATCAGTAAAGATTACGGCTATCTTACTTTTATTGTCCCTTCCTGTT
TTAGCTGGAGCTATTACCATACTTTTAACA
>JQ973041
GGACAACCCGGAACTTTACTTGGTGACGATCAACTTTATAATGTTATTGTTACTGCGCAT
GCGTTTGTAATAATTTTTTTCTTAGTTATGCCTATGATGATTGGTGGGTTTGGAAATTGA
TTAGTTCCTTTAATATTAGGAGCTCCAGACATAGCTTTTCCTCGGTTAAATAATATGAGT
TTTTGACTTTTACCGCCTGCTCTTCTTTTATTACTTTCTTCAGCTGCAGTTGAGAGTGGT
GTGGGAACAGGATGAACTGTTTATCCTCCTTTATCTGCAAATCTGGCTCATGCTGGTGGT
TCTGTAGATCTTGCAATTTTTTCTTTACATTTAGCAGGTGCTTCTTCTATTTTAGGTGCT
GTAAACTTTATTACTACAGTTATTAACATACGTTGAGGGGGTATACCACTCGAACGGCTT
CCTCTTTTTGTTTGATCAGTAAAGATTACGGCTATCTTACTTTTATTGTCCCTTCCTGTT
TTAGCTGGAGCTATTACCATACTCTTAACA
>KR870998
GGACAACCCGGAACTTTACTTGGTGACGATCAGCTTTATAATGTTATTGTTACTGCGCAT

GCGTTTGTAATAATTTTTTTCTTAGTTATGCCTATGATGATTGGTGGGTTTGGAAATTGA
CTAGTTCCTTTAATATTAGGGGCTCCAGACATAGCTTTTCCTCGGTTAAATAATATAAGT
TTTTGACTTTTACCGCCTGCTCTTCTTTTATTGCTTTCTTCAGCTGCAGTTGAAAGTGGC
GTGGGAACAGGATGAACTGTTTATCCTCCTTTATCTGCAAATCTGGCTCATGCTGGTGGT
TCTGTAGATCTTGCAATTTTTTCTTTACATTTAGCAGGTGCTTCTTCTATTTTAGGTGCT
GTAAACTTTATTACTACAGTTATTAACATACGTTGAGGGGGTATGCCACTTGAACGGCTT
CCTCTCTTTGTTTGATCAGTAAAGATTACGGCTATCTTACTTTTATTATCCCTTCCTGTT
TTAGCTGGAGCTATTACCATACTCTTAACA
>KR870995
GGACAACCCGGAACTTTACTTGGTGACGATCAGCTTTATAATGTTATTGTTACTGCGCAT
GCGTTTGTAATAATTTTTTTCTTAGTTATGCCTATGATGATTGGTGGGTTTGGAAATTGA
CTAGTTCCTTTAATATTAGGAGCTCCAGACATAGCTTTTCCTCGGTTAAATAATATGAGT
TTTTGACTTTTACCGCCTGCTCTCCTTTTATTACTTTCTTCAGCTGCAGTTGAGAGTGGT
GCGGGAACAGGATGAACTGTTTATCCTCCTTTATCTGCAAATCTGGCTCATGCTGGTGGT
TCTGTAGATCTTGCAATTTTTTCTTTACATTTAGCAGGTGCTTCTTCTATTTTAGGTGCT
GTAAACTTTATTACTACAGTTATTAACATACGTTGAGGGGGTATGCCACTTGAACGGCTT
CCTCTCTTTGTTTGATCAGTAAAGATTACGGCTATCTTACTTTTATTATCCCTTCCTGTT
TTAGCTGGAGCTATTACCATGCTCTTAACA
>JQ973021
GGACAACCCGGAACTTTACTTGGTGACGATCAGCTTTATAATGTTATCGTTACTGCACAT
GCGTTTGTAATAATTTTTTTCTTAGTTATACCTATAATGATCGGAGGATTTGGAAATTGG
TTGGTTCCTTTAATGTTAGGAGCCCCAGACATAGCTTTTCCTCGATTAAATAATATAAGT
TTTTGATTACTACCGCCTGCCCTTCTTTTATTACTTTCTTCAGCTGCAGTTGAAAGTGGT
GTGGGGACGGGATGAACTGTTTATCCTCCTCTATCTGCGAATCTAGCTCATGCTGGTGGT
TCTGTAGATCTTGCAATTTTTTCTTTACATTTAGCAGGTGCTTCTTCTATTTTAGGTGCT
GTAAATTTTATTACTACGGTTATTAATATACGTTGAGGGGGTATACCACTTGAACGACTT
CCTCTTTTTGTCTGATCGGTAAAAATTACAGCTATTTTACTTCTATTGTCTCTTCCTGTT
TTAGCTGGAGCTATTACTATACTTTTAACA
>KM220906
GGACAGCCTGGAACTCTATTAGGTGACGATCAGCTTTATAATGTTATTGTTACTGCCCAT
GCATTTGTAATAATTTTCTTTTTAGTTATACCTATGATGATTGGTGGATTTGGAAATTGA
TTGGTTCCTTTAATATTAGGGGCTCCAGACATGGCTTTTCCTCGATTAAATAATATAAGT
TTTTGGTTACTTCCGCCTGCTCTTCTTTTATTACTTTCTTCTGCAGCTGTTGAAAGTGGT
GTTGGGACTGGGTGAACTGTTTATCCTCCGTTATCTGCAAATATTGCTCATGCTGGTGGC
TCAGTAGATCTTGCAATTTTTTCTTTACATTTAGCTGGTGCTTCATCTATTTTAGGGGCT
GTAAATTTTATTACTACAATTATTAATATACGTTGACGAGGGATGCCACTTGAACGTCTT
CCGTTGTTTGTATGGTCTGTAAAAATTACAGCTATTTTACTTCTTTTATCCCTTCCAGTT
TTGGCTGGAGCTATTACAATGCTTCTTACA
>AF129322
GGGCAACCTGGGACTTTACTTGGTGACGATCAGCTTTATAATGTTATTGTGACTGCACAT
GCGTTTGTAATAATTTTTTTCTTAGTTATGCCCATAATAATTGGCGGGTTTGGAAATTGA
TTAGTTCCTTTAATATTAGGGGCTCCAGACATAGCTTTTCCTCGGTTAAATAATATAAGT
TTTTGATTATTACCACCTGCTCTTCTTTTATTACTTTCTTCAGCTGCAGTTGAGAGCGGT
GTAGGTACAGGATGAACTGTTTATCCTCCTTTATCTGCAAATCTAGCTCATGCTGGAGGT
TCTGTAGATCTTGCGATTTTTTCTTTACACTTAGCAGGTGCTTCTTCTATTTTAGGTGCT
GTAAATTTTATTACTACAGTTATTAATATACGTTGAGGAGGTATGCCACTTGAACGGCTT
CCTCTTTTTGTTTGATCAGTAAAGATTACGGCTATTTTACTTTTATTATCTCTTCCTGTC
TTAGCTGGAGCTATTACTATGCTTTTAACA
